# Treatment and Management of Upper Extremity Dysfunction Following Transradial Percutaneous Coronary Intervention: A Prospective Cohort Study

**DOI:** 10.1177/15589447211073832

**Published:** 2022-03-04

**Authors:** Elena S. Cheung, Eva M. Zwaan, Ton A. R. Schreuders, Marcel J. M. Kofflard, J. Henk. Coert, Marco Alings, Alexander J. J. IJsselmuiden, Carlo A. J. Holtzer

**Affiliations:** 1 University Medical Center Utrecht, Utrecht, The Netherlands; 2 Amphia Hospital, Breda, The Netherlands; 3 Erasmus Medical Center, Rotterdam, The Netherlands; 4 Albert Schweitzer Hospital, Dordrecht, The Netherlands

**Keywords:** coronary angiography, hand rehabilitation, percutaneous coronary intervention, referral, transradial access

## Abstract

**Background:**

The transradial artery access is the benchmark approach in transradial percutaneous coronary intervention (TR-PCI). The purpose of this study was to evaluate the different complications, treatments, and outcome of upper extremity dysfunction following a TR-PCI.

**Methods:**

This was a prospective cohort substudy of patients with access-site complications. The study population consisted of 433 patients treated with TR-PCI. Referral to the hand center was mandated if the patient experienced new-onset or increase of preexistent symptoms in the upper extremity. Patients were followed up to the last control visit (5-7 months after the index procedure) at the hand center. Outcome results were categorized in “symptom-free,” “improvement of symptoms,” and “no improvement.”

**Results:**

Forty-one (9% of total) patients underwent assessment at the hand center. Most frequent referral indication was pain in the intervention arm. Women, preexisting sensibility disorder, and osteoarthritis in the intervention arm were associated with increased odds of referral. The most common complications diagnosed were carpal tunnel syndrome (n = 18) and osteoarthritis (n = 15). Thirty patients required further medical treatment. Immobilization therapy was most applied. Seventeen (4% of total) patients had persisting symptoms despite medical treatment.

**Conclusions:**

The occurrence of complications in the upper extremity after a TR-PCI is small. Despite medical treatment, symptoms persisted in 4% of all patients treated with TR-PCI. Possible explanations for the persisting symptoms are exacerbation of latent osteoarthritis and carpal tunnel syndrome by trauma-induced edema. Awareness of TR-PCI-induced complications among all specialists is essential to optimize patient care.

## Introduction

Transradial percutaneous coronary intervention (TR-PCI) has become the gold standard in treating coronary artery stenosis.^1^ In Europe, approximately 1.8 million PCIs are performed each year, of which more than two-thirds via the transradial approach. Transradial PCI-related upper extremity dysfunction (UED) might cause minor and short-term sequelae, as well as lifelong.^2^

Compared with femoral access, in patients with ST-segment elevation myocardial infarction, radial access caused 73% less major access bleeding, with a trend toward reduction of mortality and ischemic events.^3^ Upper extremity dysfunction following TR-PCI has not been properly investigated.^4^ The *Effects of trAnsRadial perCUtaneouS coronary intervention on upper extremity function* (ARCUS) study is a multicenter prospective cohort study, providing insight in access-site complications and morbidity after TR-PCI. An interim analysis of the ARCUS study at 2 weeks of follow-up showed that 63% of patients had manifestations of UED according to the ARCUS criteria (Table 1). The main complaint that would lead to referral to a hand center was found to be pain.^5^

**Table 1.** Composed Score for the Occurrence of Upper Extremity Dysfunction.

Upper extremity dysfunction at follow-up vs baseline
Increased NRS of ≥2 points
Absent signal of the radial artery during Doppler ultrasound examination.
Strength:
≥15% decrease in palmar grip strength ≥15% decrease in key grip strength ≥15% decrease in isometric strength of flexion and extension of the elbow and wrist
≥2 filament increase in sensibility of the hand according to the WEST
≥2-cm increase of circumference of the hand
≥2-cm increase of circumference of the forearm

*Note*. Positive score (dysfunction present) if at least 2 of the criteria were present. Pain is measured using NRS (score: 0-10, 0 = no pain). NRS = Numeric Rating Scale; WEST = Weinstein Enhanced Sensory Test.

We hypothesize that access-site complications in the upper extremity following TR-PCI can persist, even after targeted treatment. The main objective of this study is to investigate access-site complications, referral rates to hand centers, and treatment outcomes of UED after TR-PCI.

## Materials and Methods

In a substudy of the ARCUS cohort study, treatment and management of UED within 6 months after a TR-PCI was assessed in ARCUS individuals who were referred to hand centers. Patients were enrolled in 2 high-volume centers in the Netherlands between 2014 and 2019.

### Inclusion Criteria

Patients were included if they had a palpable radial artery, Doppler ultrasound examination of the artery confirmed nonocclusive flow, and were subsequently treated with TR-PCI. Exclusion criteria were clinical conditions that prevented patients from giving informed consent and/or taking the baseline examination of both arms. Patients were also excluded if they had progressive musculoskeletal diseases or other comorbidities that could limit their ability to participate in the study or to comply with follow-up requirements.^6^

### Measurements and Recordings

In the ARCUS study, patients were examined for the presence of UED at baseline, 2 weeks, 1 month, and 6 months of follow-up. Investigations were identical for all follow-up moments. An examination consisted of physical tests and validated questionnaires. Presence of UED was defined as greater than or equal to 2 criteria at follow-up (Table 1). Hand function was examined using goniometry of the upper extremity, noted in degrees; the Kapandji thumb opposition scores,^7^ with scores presented on a 11-point scale (0: no opposition, 10: maximal opposition),^8^ manual muscle strength measurement of the thenar muscles ranked according to the Medical Research Council scale, and assessment of the tactile sensation using the Weinstein Enhanced Sensory Test,^9^ with results presented on a 5-point scale. Palmar grip and key pinch strength measurements were taken in the standardized position: standing with the arm held flush to the side of body, elbow flexed to 90°, forearm in the mid-prone position, and wrist in the neutral position, using a Jamar hydraulic hand dynamometer and Jamar hydraulic pinch gauge (Jamar, Patterson Medical, Illinois).^10,11^ Isometric strength of flexion and extension of the elbow and wrist were assessed using a microFET2 digital handheld dynamometer (microFET2, Utah).^12^ Volume of the hand was measured using a standardized figure-of-eight method,^13^ whereas volume of the forearm was measured circumferentially 8 cm distal of the medial epicondyle,^14^ both noted in centimeters. The functional status was assessed using a validated questionnaire: the Numeric Rating Scale (NRS) for pain.^15^

All patients were treated with conventional 6-French (6F) TR-PCI followed by nonpatent hemostasis of the radial artery using a compression device. The compression device was applied for a duration of 24 hours after completion of the procedure. Patients were advised to minimize manual activity after procedure.

Procedure-specific information, such as pain during procedure, duration of procedure, operator, materials used, and total number of punctures, was collected from the ARCUS database.

### Referral

Two groups of patients were distinguished: referred patients and nonreferred patients. New-onset or increase of preexistent symptoms in the upper extremity mandating referral were defined as pain objectified using the NRS, paresthesia (numbness and/or tingling), loss of strength, swelling, major access-site hematoma accompanied by pain, atrophy of muscle, blood flow disorders, and symptomatic occlusion (pain or blood flow disorders) of the radial artery.

### Follow-up

Relevant follow-up data from the hand center were collected from the medical charts. Relevant data from the hand center were reason for referral, physical examination, diagnostic tools, diagnosis, date of treatment, treatment given, and treatment outcome.

### Endpoints

The primary study endpoint was defined as the percentage of patients with persisting symptoms in the upper extremity at the last control visit (5-7 months after the index procedure) to the hand center. Treatment results were categorized in “symptom-free after treatment,” “improvement of symptoms after treatment,” and “no improvement after treatment.” Secondary endpoints were to examine possible risk factors for referral using multiple logistic regression and referral correlation with upper extremity measurements as defined in the ARCUS study (Table 1).

### Statistical Methods

For baseline characteristics, data of the categorical variables were presented as percentages. Continuous variables with normal distribution were described in terms of mean and standard deviation, and continuous variables who were not normally distributed were described in terms of medians and interquartile range. Between-group differences were analyzed using the Pearson χ^2^ test for categorical variables and phi correlation categorized in weak (−0.3 to −0.1 or 0.1 to 0.3), moderate (−0.5 to −0.3 or 0.3 to 0.5), and strong (−1.0 to −0.5 or 1.0 to 0.5); the independent *t* test for normally distributed variables; and the Mann-Whitney *U* test for continuous variables that were not normally distributed.

Multivariable logistic regression was performed with having a referral indication as the outcome variable and independent factors (patient characteristics, history of UED, and procedure specifications) as predictor variables. Independent factors were evaluated in a univariate analysis, where variables with a value of *P* < .30 were identified as potential predictors. Normality of the distributions of numerical independent variables was assessed statistically and graphically. Multicollinearity between independent factors was examined using tolerance, variance inflating factors (VIF), and a correlation matrix. Variables with a tolerance level of less than 0.10 and/or a VIF of greater than 10 were excluded from the final regression model. The final model was built using the “Backward stepwise” regression method. Results were presented as odds ratios with 95% confidence intervals.

Pearson χ^2^ test with continuity correction was used to compare individual measurements (Table 1) between referral and nonreferral patients. Referral indication, diagnostics, and treatment results were represented using tables. To analyze the necessity of different treatments in the respective arms (intervention upper extremity, nonintervention upper extremity, and bilateral arms), the Cochran Q test was used. McNemar test for paired proportions was used to compare the occurrence of diagnosis in the intervention arm versus the nonintervention arm in each referred patient. The primary endpoint was defined as the percentage of patients with persisting symptoms in the upper extremity at the last control visit. Individuals with missing values were excluded from the specific outcome measure analysis. Time until treatment was presented in mean months with standard deviation and analyzed using Kruskal-Wallis test.

All relevant data were collected using Microsoft Excel (Microsoft, Redmond, Washington) and OpenClinica (Waltham, Massachusetts). Analyses were conducted using SPSS for Mac version 26 (SPSS Inc, Chicago, Illinois). All statistical tests used a statistical significance level of 5%.

### Ethical Considerations

Informed consent was obtained from all individual participants included in the study. The ARCUS study protocol was approved by local and regional ethics committees (Toetsingscommissie wetenschappelijk onderzoek Rotterdam, Rotterdam, the Netherlands), in accordance with the Declaration of Helsinki. All patients provided written informed consent before the study.

## Results

### Patient Characteristics

**Figure 1.**
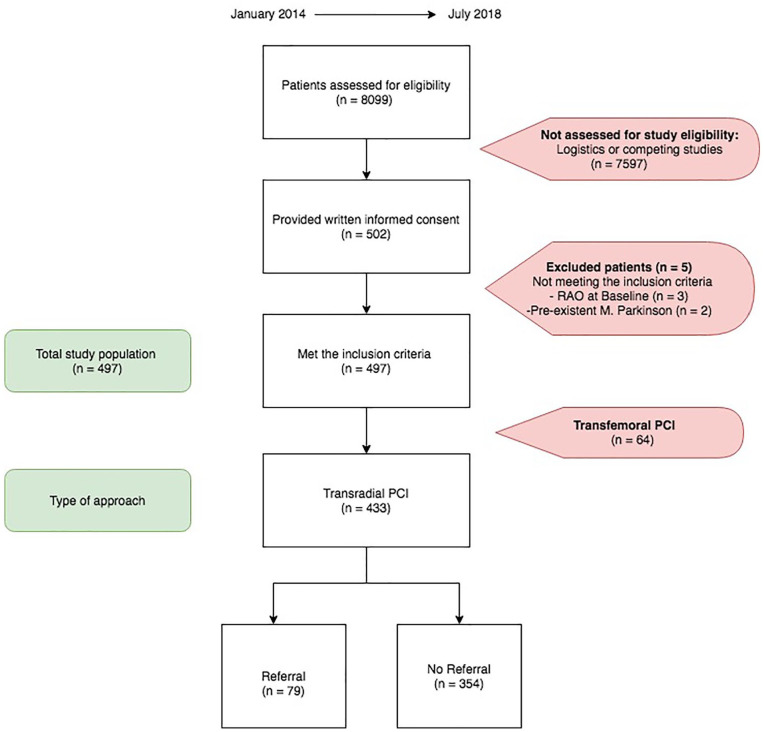
Study flow chart. *Note*. PCI = percutaneous coronary intervention; RAO = radial artery occlusion.

The ARCUS study included 433 patients (from January 2014 to January 2019) who underwent TR-PCI. Seventy-nine patients (18%) fulfilled the criteria for referral to a hand center (referral group) (Figure 1, Table 2).

**Table 2.** Baseline Demographic and Clinical Characteristics.

Parameter	Referral (n = 79)	Non-referral (n = 354)	*P* value^a^
Age, mean (SD)	63.9 (9.4)	65.7 (10.2)	.157
Female sex, no. (%)	27 (34.2)	69 (19.5)	.007
Body mass index, mean (SD)	29.1 (4.6)	27.6 (4.3)	.005
Smoking status, no. (%)			.326
Active	9 (11.4)	63 (17.8)	
Never	29 (36.7)	110 (31.1)	
Stopped	41 (51.9)	181 (51.1)	
Medical history, no. (%)			
Diabetes mellitus	15 (19.0)	80 (22.6)	.582
Hyperlipidemia	35 (44.3)	140 (39.5)	.514
Hypertension	47 (59.5)	195 (55.1)	.556
Osteoarthritis intervention arm	25 (31.6)	38 (10.7)	<.001
Osteoarthritis non-intervention arm	21 (26.6)	38 (10.7)	<.001
CTS intervention arm	8 (10.1)	24 (6.8)	.429
CTS non-intervention arm	10 (12.7)	24 (6.8)	.127
Sensibility disorder intervention arm	18 (22.8)	15 (4.2)	<.001
Sensibility disorder non-intervention arm	14 (17.7)	26 (7.3)	.008
Polyneuropathy	4 (5.1)	7 (2.0)	.238
Right hand dominance, no. (%)	69 (87.3)	316 (89.3)	.769
Employed, no. (%)	24 (30.4)	110 (31.1)	1.00
Previous TR-PCI, no. (%)	27 (34.2)	102 (28.8)	.420
Crossover to femoral artery, no. (%)	8 (10.1)	19 (5.4)	.185

*Note*. CTS: carpal tunnel syndrome; TR-PCI: transradial percutaneous coronary intervention.

a χ^2^ test for categorical variables, and parametric *t* test (mean) for continuous variables.

There was an association identified between referral and higher mean body mass index, female sex, preexistent changed sensation, and preexistent osteoarthritis in both extremities (Table 2). However, there was no association between referral and age (*P* = .120) (Table 3).

**Table 3.** Symptoms Leading to a Referral to the Hand Center.

Symptom	Intervention arm	Nonintervention arm	*P* value^a^
Pain, no. (%)	55 (69.6)	17 (21.5)	<.001
New-onset	43 (78.2)	13 (76.5)	
Progressive	12 (21.8)	4 (23.5)	
Loss of strength, no. (%)	34 (43.0)	11 (13.9)	<.001
New-onset	30 (88.2)	9 (81.8)	
Progressive	4 (11.8)	2 (18.2)	
Changed sensation, no. (%)	47 (59.5)	23 (29.1)	<.001
New-onset	37 (78.7)	19 (82.6)	
Progressive	10 (21.3)	4 (17.4)	
Blood flow disorder, no. (%)	7 (8.9)	4 (5.1)	.25
New-onset	7 (100.0)	4 (100.0)	
Progressive	-	-	
Occlusion of the radial artery, no. (%)	7 (8.9)	0 (0.0)	-

a McNemar test for paired proportions was used to compare the occurrence of symptoms in the intervention arm versus the nonintervention arm in each referred patient.

### Referral Symptoms and Predictors

Pain was most frequently reported as the reason for referral (Table 3). New-onset or progressive pain, loss of strength, paresthesia, and absent Doppler signal of the radial artery were all significantly more present in the intervention arm (Table 3). Multivariate logistic regression analysis was performed to identify baseline and/or procedural factors that independently could predict clinically relevant upper extremity complaints following TR-PCI (Table 4). The logistic regression model with 7 variables, χ^2^ (7, N = 388) = 48.556, *P* < .001, was able to distinguish between respondents who did and did not have clinically relevant complaints of the upper extremity. The model explained between 11.8% (Cox and Snell) and 19.2% (Nagelkerke *R*^2^) of the variance in referral status, and correctly classified 84.0% of cases. Variables that significantly increased the odds for a referral were female sex, osteoarthritis of the intervention upper extremity, and sensibility disorders of the intervention upper extremity. Use of oral anticoagulants and higher age decreased the odds for referral (Table 4).

**Table 4.** Multiple Logistic Regression Analysis Predicting Likelihood of a Referral Indication.

Covariates^a^	*B*	SE	*P* value	Odds ratio	95% CI
Constant	−0.235	1.483	.881	0.802	
Female	0.668	0.331	.044	1.951	1.019-3.736
Age	−0.030	0.015	.040	0.970	0.942-0.999
Body mass index	0.051	0.031	.098	1.053	0.991-1.119
Caregiver present	−0.813	0.461	.078	0.444	0.180-1.095
Sensibility disorders intervention arm	1.116	0.442	.012	3.051	1.284-7.254
Osteoarthritis intervention arm	0.799	0.378	.034	2.223	1.060-4.662
Oral anticoagulants^b^	−0.749	0.296	.011	0.473	0.265-0.845
Test	χ^2^	*df*	*P* value		
Overall model evaluation					
Likelihood ratio test	48.56	7	<.001		
Goodness-of-fit test					
Hosmer and Lemeshow	9.84	8	.276		

*Note*. CI = confidence interval; LMWH= low-molecular-weight heparin.

a Baseline characteristics.

b Oral anticoagulants, for example, acenocoumarol, clopidogrel, LMWH, and heparin.

### Individual Measurements Between Patients With and Without Referral

At all follow-up moments, referral was associated with a clinically important increase in NRS pain score in the intervention upper extremity only (2-week follow-up) or in both extremities (1- and 6-month follow-up). The proportion between referral and nonreferral patients with a clinically important increase in pain score was higher in the intervention arm (Table 5).

**Table 5.** Physical Measurements Between Patients With and Without Referral.

Measurement	Criteria^a^	Referral (%)	Nonreferral (%)	*P* value^b^
2 Weeks				
NRS pain score intervention arm	≥2	48.1	7.20	<.001
Deviant Doppler intervention arm	Absent signal	11.1	0.92	.002
1 Month				
NRS pain score intervention arm	≥2	40.9	5.97	<.001
NRS pain score nonintervention arm	≥2	26.1	4.52	<.001
Extension wrist in the nonintervention arm	≥15% decrease	54.5	9.78	<.001
Palmar grip intervention arm	≥15% decrease	22.7	6.12	.013
6 Months				
NRS pain intervention arm	≥2	58.3	8.41	<.001
NRS pain nonintervention arm	≥2	37.5	7.49	<.001
Deviant Doppler intervention arm	Absent signal	12.5	1.22	.002

*Note*. Each measurement was examined in the intervention and nonintervention arm. NRS = Numerical Rating Scale.

a Criteria defined as in the ARCUS study, see Table 1.

b χ^2^ test for categorical variables.

### Assessment at Hand Centers

Only 41 patients (52%) of the referral group were seen by a hand specialist and were subsequently diagnosed with at least 1 complication. Thirty-eight patients choose to refrain from further assessment, indicating not clinically relevant complaints. The most frequent reason for refraining from further assessment was a watchful waiting policy (n = 18, 47%), followed by unwillingness to undergo further assessment (n = 15), personal circumstances (n = 4), and when patients considered a referral unnecessary (n = 1).

### Diagnosis and Treatment

Carpal tunnel syndrome (CTS) was diagnosed in 18 patients: bilateral CTS in 13 patients and unilateral CTS in the intervention arm in 5 patients (Table 6). Fourteen patients had new-onset CTS after TR-PCI. Most patients diagnosed with CTS were men (56%). Five patients were managed with observation and 13 patients received additional treatments. Ten patients required carpal tunnel release (not related to hematoma), of which 50% with additional hand therapy or injection. The other 2 patients were managed with injections or hand therapy. In 7 patients, symptoms had resolved at the last follow-up, 3 patients experienced a decrease of symptoms, 2 patients experienced no improvement, and 1 patient experienced an increase in symptoms.

**Table 6.** Complications Diagnosed After a TR-PCI.

Complication, no.	n
Carpal tunnel syndrome	18
Osteoarthritis	15
Major hematoma	5
Arterial perforation	1
Tendinitis	3
Ulnar neuropathy	2
Symptomatic radial artery occlusion	7
Wartenberg syndrome	1

*Note*. TR-PCI = transradial percutaneous coronary intervention.

Fifteen patients were diagnosed with osteoarthritis: unilateral osteoarthritis in the intervention arm in 8 patients and bilateral osteoarthritis in 7 patients. Osteoarthritis was significantly more common in the intervention upper extremity than in the nonintervention upper extremity (37% vs 17%, *P* = .008). Eight patients had progression of preexistent osteoarthritis and 7 patients did not have a history of osteoarthritis in the upper extremity. Thumb base (CMC-1 joint) osteoarthritis was diagnosed in 10 patients, wrist osteoarthritis in 1 patient, and both thumb base (CMC-1 joint) and wrist osteoarthritis in 3 patients. Three patients were managed with observation and 12 patients received hand therapy and/or immobilization treatment. One patient underwent additional proximal row carpectomy combined with synovectomy of the radial extensor tendon. In 4 patients, symptoms had resolved at the last follow-up, 7 patients experienced a decrease of symptoms, and 1 patient experienced no improvement.

Five patients were diagnosed with (major) hematoma on the day of the procedure: 1 patient with a brachial artery perforation and 4 patients with a local hematoma in the wrist or forearm with swelling. All were treated nonsurgically: use of compression wrap/sleeve, compression with a TR-band, or elevation with a sling. No residual complications were noted during the follow-up period.

Tendinitis was diagnosed in 3 patients: 2 patients with flexor carpi radialis tendinitis and 1 patient with Morbus de Quervain tendinitis. All 3 patients were managed with immobilization therapy. At the end of the follow-up, 2 patients had resolution of symptoms and 1 patient experienced no improvement in symptoms.

Four patients were diagnosed with unspecified pain symptoms following TR-PCI. Three patients were treated with observation only and 1 patient with hand therapy resulting in a decrease in symptoms.

Symptomatic radial artery occlusion (RAO) was diagnosed in 7 patients at the time of referral (Table 3). Four patients declined further assessment. The 3 remaining patients were managed conservatively.

Two patients were diagnosed with ulnar neuropathy. One patient was managed conservatively and experienced a decrease in symptoms. One patient was treated with surgical decompression of the ulnar nerve and cortisone injection. This did not result in an improvement of symptoms.

One patient was diagnosed with Wartenberg syndrome and was treated with surgical decompression of the superficial radial nerve. Symptoms remained unchanged at the end of follow-up.

### Treatment Outcome

In total, 17 of the 30 treated patients (57%) experienced persisting symptoms, at mean 5 months (SD, 4) after the transradial procedure: 76% experienced a decrease of symptoms, 18% experienced no improvement, and 6% experienced an increase in symptoms. Most patients with persisting symptoms received treatment for osteoarthritis or CTS in either the intervention arm only or in both arms (13 of the 17 patients). Fourteen patients (14 of the 17 patients) with persisting symptoms were newly diagnosed with CTS or osteoarthritis: 6 patients were diagnosed with CTS of which 1 patient had a history of CTS. Eight patients were diagnosed with osteoarthritis of which 2 patients had a history of osteoarthritis. There was no significant difference in time between the TR-PCI procedure and start of treatment for UED across the 4 outcome categories (*P* = .24).

## Discussion

This study offers insight into the spectrum of complications in the upper extremity after a TR-PCI, as well as the treatment and clinical outcome. The follow-up time of 6 months enabled us to eliminate the effect of an overall impaired condition related to the underlying cardiac pathology. Despite medical treatment, symptoms persisted in 4% of patients undergoing TR-PCI. For the European interventions, this would suggest that nearly 50 000 patients each year have persistent symptoms after a TR-PCI.

Symptomatic RAO had a prevalence of 9% in the intervention upper extremity (Table 3). This is in line with other studies, where symptomatic and asymptomatic RAO were the most common complication with a prevalence of 1% to 10%.^16,17^ Because RAO prohibits future transradial access, and radial artery usage for coronary artery bypass grafting, this complication should be prevented.

Women were more likely to develop a referral indication following TR-PCI. An explanation might be that in general, women have a smaller radial artery diameter than men, which then results more frequently in a mismatch in sheath-to artery ratio with the conventional 6F-sized catheters.^18^

Carpal tunnel syndrome was the most frequent diagnosed. In our study, 18 (4% of 433) patients had CTS in the intervention hand, similar to the prevalence in the general population (4%).^19^ Carpal tunnel syndrome is caused by entrapment of the median nerve, and a dose-response curve exists between duration and amount of pressure and median nerve damage.^20,21^ Although it is improbable that the occurrences of CTS were solely caused by the transradial procedure, it seems likely that the functional response to prolonged application of a radial artery pressure device after procedure, as well as trauma-induced edema contributed to the development of CTS, or exacerbated preexisting CTS.^21^ Other explanations could be a trauma-induced hematoma, swelling of the wrist caused by inflammation, or ischemic vascular injury caused by compression.^21,22^

Patients with a history of hand osteoarthritis in the intervention arm were more likely to develop a referral indication. In our study, most patients diagnosed with osteoarthritis were diagnosed with exacerbation of osteoarthritis. Osteoarthritis is a degenerative disease; however, TR-PCI might provoke or exacerbate latent osteoarthritis through inactivity of the joint following the procedure.^23^

We found a higher incidence of complications than a recent study investigating acute complications following transradial catheterization.^24^ In accordance with our results, complications occurred more frequently in women. However, CTS was diagnosed in only 4 of the 10 540 patients in the same study compared with 4% in our study.^24^ Moreover, (exacerbation of) osteoarthritis was not recognized as a complication of TR-PCI.^24^ It is likely that the retrospective design of the mentioned study resulted in an underestimation of complications.

A unique quality of this study is that functional outcomes could be compared between referral and nonreferral patients (Table 5). Most significant functional differences pertained to the intervention arm; however, we also found a significant decrease in strength in the nonintervention arm (Table 5). The considerable number of patients with complaints in the nonintervention upper extremity can be explained by an increased focus and awareness regarding the upper extremity function or impaired overall condition caused by physical inactivity following the cardiac procedure.

Our study has several clinical implications. First, awareness among cardiologists and hand specialists regarding UED following TR-PCI must be improved. Most PCI patients in Europe are treated via the transradial approach; therefore, postprocedure UED could affect a large group of patients. Cardiologists mostly focus on the cardiac result of TR-PCI only, neglecting the severity of procedure-related UED. Our result showed that UED could persist up to 6 months.

Furthermore, the relatively high percentage of patients that refrained from further assessment may be due to the fact that patients experience complications in the upper extremity as a minor problem compared with their underlying cardiac pathology. Efforts should be made to inform patients and to encourage appropriate follow-up in case of complications.

In our study, patients treated with conventional 6F catheters were included which benefits the generalizability of the study results. Reducing catheter size from 6F to 5 French (5F) slender and sheathless techniques might reduce incidence of complications, especially in women and older age groups.^18,25-27^ One study confirmed that in comparison with a 6F sheath, 20% more women and 10% more men complied with a sheath-to-artery ratio of less than 1 when using a 5F sheath.^27^ Radial arteries with smaller diameter may cause more spasm due to catheter friction.^26,28,29^ However, 5F PCI has several disadvantages compared with 6F PCI: a learning curve for the operator, lower trackability and visibility, less backup support, and reduced treatment options (eg, not suited for kissing balloon or kissing stenting procedures).^30^ Further research is needed on how to prevent UED following TR-PCI by minimizing procedure materials, and how to reduce morbidity of complicated TR-PCI.

### Limitations

In this study, a cost-effectiveness analysis was not performed. Previous studies showed that, compared with femoral access, TR-PCI and transradial coronary artery angiography are associated with lower costs. However, these studies did not include treatment for post-PCI UED.^31^

Another limitation is that only ARCUS study patients were included, resulting in a limited sample size. However, comparison in functional status could be made with the ARCUS measurements and with the extensive database, we could control for patient factors such hand dominance, sex, and preexisting UED in a logistic regression model. Unfortunately, risk for persisting symptoms following treatment could not be controlled for, due to the limited number of referred and treated patients. No excessive constraining excluding criteria was applied in the ARCUS study, making the results generalizable to the population undergoing TR-PCI.

## Conclusion

The complication rate in the upper extremity following TR-PCI is low; however, 57% of the patients receiving targeted treatment for their complications experienced persistent symptoms. Women and patients with a history of osteoarthritis in the upper extremity were at increased risk to develop complications. Most complaints that necessitated treatment consisted of preexistent or latent CTS and osteoarthritis that were exacerbated by the intervention, possibly by local edema or immobilization. Awareness of TR-PCI-induced complications among hand specialists is essential to optimize patient care.
